# Dissatisfaction with body image and weight gain in middle-aged women: A cross sectional study

**DOI:** 10.1371/journal.pone.0290380

**Published:** 2024-01-11

**Authors:** Maria Socorro Medeiros de Morais, Sabrina Gabrielle Gomes Fernandes Macêdo, Rafaela Andrade do Nascimento, Mariana Carmem Apolinário Vieira, Mayle Andrade Moreira, Saionara Maria Aires da Câmara, Maria das Graças Almeida, Álvaro Campos Cavalcanti Maciel

**Affiliations:** 1 Health Sciences Center of Federal University of Rio Grande do Norte, Natal, RN, Brazil; 2 Department of Federal University of Rio Grande do Norte, Physiotherapy Natal, Natal, RN, Brazil; 3 Department of Federal University of Ceará, Physiotherapy Fortaleza, Fortaleza, CE, Brazil; University of Petra (UOP), JORDAN

## Abstract

**Objectives:**

To investigate the relationship between weight gain and body image perception in in middle-aged women.

**Methods:**

Cross-sectional study with 453 women. Body image was assessed using the Stunkard scale, in which women were classified as: satisfied or dissatisfied (general, thinness or obesity). The identification of possible factors associated with body image dissatisfaction was performed using binary logistic regression analysis.

**Results:**

The mean age was 55.7 (±9.6) years; 80.8% were classified as dissatisfied with body image. As for body composition, women satisfied with their body image had lower values of body fat and higher values of lean mass. In the logistic regression, for general dissatisfaction and obesity, the associated variables were BMI, education and physical activity. As for “dissatisfaction with thinness”, only BMI was associated.

**Conclusion:**

Thus, the prevalence of body image dissatisfaction is high in women and part of associated factors are linked to lifestyle behaviors.

## Introduction

One of the biggest milestones of women’s aging process is menopause, which corresponds to the definitive cessation of menstruation for 12 or more consecutive months, marking the end of menstrual cycles [1]. This is the result of reduced ovarian function, with a decrease in the number of follicles and various hormonal changes, being the drop in estradiol is most significant in addition to being linked to biological, mental, social and psychological changes [2], as well as body changes, such as increased deposition of adipose tissue in the central abdominal region [3].

Menopause is usually accompanied by numerous important changes in health [4], the increase in symptoms linked to it can negatively affect a woman’s perception of certain dimensions of health and social behavior [5]. It is at this stage of life that the occurrence of obesity and metabolic syndrome is three times higher when compared to previous periods [6]. One of the most evident concerns of this period is the fear of weight gain, which additionally evolves into a body that does not fit the beauty standards imposed by society, which can negatively affect the perception of body image [7,8].

In this perspective, body image was defined by Cash and Pruzinsk [9] as a multifaceted construction based on perceptual components such as the perception of physical appearance, thoughts, feelings, and attitudes about the body, which may be influenced by cultural and economic conditions of each region [10]. The assessment of body image has important implications, with a positive judgment in people with positive feelings about their appearance, fitness, or health and a negative evaluation in dissatisfied, depressed, and lonely people [11].

Body image dissatisfaction occurs when the perceived body image differs from the ideal or desired body image. This dissatisfaction is a predictor of women’s future health and requires interventions that include lifestyle changes [8,12,13].

Despite many speculations on the subject, many myths still linger and there are few studies that investigate [14,15], simultaneously, if dissatisfaction with body image and weight variations in middle-aged women are conditions associated with the hormonal changes of female aging. Therefore, it is an unavoidable event, or it is the result of self-care neglect, a sedentary lifestyle and/or low self-esteem [15].

Although there is a large literature on these two topics, the vast majority focuses on specific groups, such as adolescents, young people and pregnant women [16]. Few studies are conducted on women between 45 and 60 years of age, whose life stage is surrounded by important social, professional, and sexual changes [16].

In this sense, obesity, thinness, and body image seem to be very important problems related to menopause, being not only a medical problem but an economic and social one, due to the already known repercussions on quality of life, health and aging itself [17]. Therefore, elucidating the determinants of body dissatisfaction in this period of a woman’s life can improve the understanding and definitions of "successful aging" and help to propose interventions aimed at improving the well-being of mature women [18,19].

There are still few studies on body image, mainly in countries like Brazil. It is not known that the body image its representation changes during the course of life due to the numerous experiences experienced by women, or that it can alter the concepts that they themselves have about their own body, having important consequences for health, quality of life and negatively influencing social interaction, productivity and performance [7]. Therefore, understanding how obesity and thinness affect the perception of women during the aging process, mainly in the menopausal transition period, helps in the direction of public policies for the prevention of future damages to the public.

Therefore, the objective of the study is to investigate the relationship between weight gain and the perception of body image in middle-aged women, aiming to better study and understand female aging, focusing on active and successful aging.

## Methods

### Study design

This is a cross-sectional study carried out in two cities in Rio Grande do Norte (RN), Parnamirim and Santa Cruz, specifically in the northeast of Brazil. Parnamirim is located in the metropolitan region of Natal and has approximately 202,456 inhabitants, while Santa Cruz is located in the countryside and has a population of approximately 39,660 inhabitants. This article is a secondary analysis of a study that aimed to examine the relationship between hormone levels and functional performance in middle-aged and older women [20,21].

### Population and sample

The population consisted of women aged between 40 and 65 years, residing in the urban areas of the two mentioned municipalities and were able to travel to the assessment sites. After disseminating notices about the research in community centers and Basic Health Units (BHU), participants were recruited by convenience sampling, based on meeting the following eligibility criteria: Absence of neurological diseases, such as Parkinson’s, cerebrovascular accident or similar conditions that could compromise, in some way, data collection; present cognitive impairment identified by four or more errors in the Leganès Cognitive Test (LCT) [22]; non-smoker; or having undergone double oophorectomy surgery. In the end, 453 women were considered for the analysis.

### Procedures

All women were assessed by trained interviewers using a standardized questionnaire described below:

### Sociodemographic data

Data were collected regarding age, education, family income, marital status, and ethnicity of the participants. Age was assessed through self-report and dichotomized into “under 60 years” and “over 60 years”, while education was assessed as years of education and then dichotomized into: 0–3 years of education and over 4 years of education [21]. Family income was categorized using the Brazilian minimum wage (MW) as a reference, which, in 2014, was set at a value of R$ 788.00, with this variable being dichotomized into less than 3 MW and 3 MW or more [20].

### Anthropometric and body composition measurements

Height and weight data were collected for later calculation of the Body Mass Index (BMI). For data analysis, the older women were classified as: normal weight (BMI≤24.9 kg/m^2^) and overweight and obese (BMI≥25 kg/m^2^) [23].

Additionally, data regarding waist and hip circumference were collected using a *“fiber glass”* measuring tape, following the recommendations of the *Waist circumference and waist-hip ratio*: *report of a WHO expert consultation* [24].

Body composition was evaluated using the bioelectrical impedance (BIA) InBody R20, which automatically calculates the skeletal muscle mass (SMM) based on the equipment manufacturer’s prediction equations [25]. The assessment of BIA correlates with the predictions made by means of Dual-energy X-ray absorptiometry (DXA) [26], being considered a reliable and useful alternative for the assessment of skeletal muscle mass [27].

To calculate the lean mass and body mass weight values, the results were divided by the total weight and then multiplied by 100.

### Physical activity

For this evaluation, the volunteers were questioned about the regular practice of physical activity. Women who have involved three or more times a week for at least 30 minutes were considered as regular physical activity practitioners. For data analysis, responses were dichotomized into yes or no [20].

### Biochemical tests

Blood samples were collected by trained nurses to analyze estradiol dosage and biochemical parameters (glucose, total cholesterol, LDL, HDL, and triglycerides). After collection, the blood was sent for biochemical analysis, performed by a specialist, and the results were recorded by the professional. For the statistical analysis, the raw values and/or defined categories were considered, according to the cut-off points established in the literature [28].

### Reproductive history

Regarding reproductive history, four variables were considered: menarche age, age at first birth, parity and menopausal stage.

For menarche age, women were dichotomized according to self-report into “before 13 years” and “13 years or older” [29]. Maternal age at first child was dichotomized into “before 18 years old” and “after 18 years old”, to separate those with primiparity in adolescence [20]. Parity was collected through the participants’ self-report, which was categorized into 0–2 children and 3 children or more [20].

The menopausal stage was determined using the classification stages of *STRAW* [30], being classified into three groups according to the self-report pattern of menstruation: pre-menopause (regular menstruation), perimenopause (irregular menstruation, with a difference in cycle duration greater than seven days up to one year of amenorrhea) or post-menopause (absence of menstruation for more than a year).

### Body image perception

For this assessment, the Stunkard scale was used, which has been widely used in epidemiological studies since it is practical, quick, and easy to apply [31] and has been validated for Brazilian women [32].

This scale consists of drawings with different human shapes, numbered from 1 to 9, with the first silhouette being the thinnest and the ninth the most obese. The set of silhouettes was presented to the women, who chose two of them: the one that best represented their current physical appearance and the one they would like to have. The scale score was given by the result of the difference in the number obtained between the current body silhouette and the desired silhouette.

For data analysis, the woman whose difference score were equal to zero was considered “satisfied with her body image”. Any other score indicates that the woman was “dissatisfied with her body image” [7]. When the difference was positive (>0), women were classified as having "dysfunction due to obesity", whereas when the difference was negative, women presented "dysfunction due to thinness" (<0). For this study, thinness is understood as BMI≤24.9 kg/m^2^ and overweight/obesity when ≥25 kg/m^2^).

### Ethical aspects

The present study was approved by the Research Ethics Committee of the Federal University of Rio Grande do Norte, with opinion no. 1.875.802. All volunteers were informed, verbally and in writing, about the research objectives and procedures and at the first contact they signed the Informed Consent Form (ICF), in accordance with resolution 510/2016 of the National Health Council.

### Statistical analysis

Data were analyzed using the statistical program Statistical Package for Social Sciences (SPSS), version 20.0 (SPSS, Chicago, IL, USA). Initially, the normality of the data was checked using the Kolmogorov-Smirnov test, then descriptive statistics were performed. Means and standard deviations for the quantitative variables, and absolute and relative frequencies for the categorical variables were used, according to the categories of body perception.

Student’s t test was used to verify the relationship between the categories of body perception and the other covariates. Then, three logistic regression models were performed to identify predictors of general body, thinness, and obesity dissatisfaction. All models were adjusted by covariates, which showed p<0.20 in the bivariate analysis, with only the variables with statistical significance remaining in the final models. In all tests, a significance level or p value < 0.05 and 95% confidence intervals were used.

## Results

The sample consisted of 453 women with an average age of 54.9 (±10.01) years, of which only 19.2% reported satisfaction with their body image, while 80.8% were dissatisfied, with 4.6% for thinness and 76.2% for obesity. When comparing women who reported body satisfaction, it can be observed that women who considered themselves dissatisfied with their image have a lower average age (54.96±9.14 vs 59.11±11.08; p-value = 0.02), lower education (85% vs 36.8%; p <0.001), a higher proportion of overweight and obesity (86.9% vs 59.8%; p <0.001) and not practice physical activity (66.9% vs 49.4%; p-value = 0.02). The other characteristics of the sample, in addition to the results of the analyzes between the groups of satisfied and dissatisfied women, are described in Table 1.

**Table 1:** Sample characterization and analysis of covariates regarding body image (n = 453), Parnamirim and Santa Cruz, 2017.

Variables	Satisfied (n = 87) Mean (±DP) or n (%)	Dissatisfied (n = 366) Mean (±DP) or n (%)	n (%) * Total	p-value
**Age (in years)**	59.14 (11.08)	54.96 (9.14)	55.76 (9.67)	**0.001** ^**a**^
**Age**				
< 60 years	42 (48.3)	100 (27.3)	142 (31.3)	**<0.001** ^**b**^
≥ 60 years	45 (51.7)	266 (72.7)	311 (68.7)	
**Education**				
0–3 years	32 (36.8)	311 (85.0)	366 (80.8)	**<0.001** ^**b**^
4 years or more	55 (63.2)	55 (15.0)	87 (19.2)	
**Family income**				
≥ 3MW	24 (27.6)	110 (30.1)	134 (29.6)	0.64 ^**b**^
< 3 MW	63 (72.4)	256 (69.9)	319 (70.4)	
**Ethnicity**				
White	32 (36.8)	119 (32.5)	151 (33,3)	0.48 ^**b**^
Brown/Black	55 (63.2)	247 (67.5)	302 (66,7)	
**Marital status**				
No	26 (29.9)	109 (29.8)	140 (30.9)	0.97 ^**b**^
Yes	61 (70.1)	257 (70.2)	313 (69.1)	
**BMI**				
Normal	35 (40.2)	48 (13.1)	83 (18.3)	**<0**.**001** ^**b**^
Overweight and Obese	52 (59.8)	318 (86.9)	370 (81.7)	
**Menopausal Stage**				
Pre-menopause	13 (14.9)	81 (22.2)	94 (20.8)	0.32 ^**b**^
Perimenopause	12 (13.8)	45 (12.3)	57 (12.6)	
Post-menopause	62 (71.3)	240 (65.5)	302 (66.6)	
**Physical activity**				
No	43 (49.4)	245 (66.9)	288 (63.6)	**0.02** ^**b**^
Yes	44 (50.6)	121 (33.1)	165 (36.4)	
**Early maternal age**				
No child	6 (6.9)	13 (3.6)	19 (4.2)	0.28 ^**b**^
<18 years old	20 (23.0)	74 (20.2)	94 (20.8)	
18 years or older	61 (70.1)	279 (76.2)	340 (75.1)	
**Parity**				
0–2	26 (29.9)	147 (40.2)	173 (38.2)	0.07 ^**b**^
3 or more	61 (70.1)	219 (59.8)	280 (61.8)	
**Menarche**				
Before age 13	40 (46.0)	147 (40.2)	187 (41.3)	0.32 ^**b**^
13 years or older	47 (54.0)	219 (59.8)	266 (58.7)	

Notes: a: Student’s t-test p-value

b: Qui-Square test p-value

*valid n; MW: Minimum Wage; BMI: Body Mass Index.

Table 2 shows the characteristics of the sample, and the comparative analyzes of body image regarding anthropometric variables, body composition and biochemical variables. Regarding the anthropometric variables, height was the only variable that did not have a statistically significant relationship, with all other variables being related to the perception of body image, of which satisfied women had lower anthropometric values. As for body composition, women who said they were satisfied with their body image had lower values of body fat percentage (p< 0.001) and higher values of lean mass percentage (p< 0.001). Regarding biochemical variables, total cholesterol and estradiol were related to body image, where dissatisfied women had higher values in these tests.

**Table 2:** Sample characteristics and analysis of anthropometric, body composition, biochemical and body image parity variables. Parnamirim, Santa Cruz 2017.

Variables	Satisfied (n = 87) Mean (±DP) or n (%)	Dissatisfied (n = 366) Mean (±DP) or n (%)	n (%) * Total	p-value^a^
Weight	60.6 (9.04)	70.01 (12.45)	68.22 (12.42)	**<0.001**
Height	1.53 (0.06)	1.53 (0.05)	1.53 (0.06)	0.76
Waist	89.98 (8.57)	97.74 (10.44)	96.25 (10.55)	**<0.001**
Hip	100.22 (6.66)	106.18 (9.46)	105.03 (9.29)	**<0.001**
BMI	25.88 (3.41)	29.73 (4.63)	28.99 (4.67)	**<0.001**
Waist—Hip Ratio	0.89 (0.05)	0.92 (0.05)	0.91 (0.05)	**0.001**
% Body fat	37.09 (6.36)	41.69 (6.00)	40.82 (6.32)	**<0.001**
% Lean mass	62.79 (6.51)	58.25 (6.02)	59.11 (6.36)	**<0.001**
Estradiol	40.69 (64.69)	60.78 (98.45)	56.87 (93.10)	**0.04**
Glucose	95.83 (31.48)	102.32 (36.46)	100.99 (35.55)	0.14
HDL	45.52 (9.67)	46.10 (12.40)	45.97 (44.53)	0.69
LDL	121.51 (39.70)	130.57 (38.59)	128.65 (38.95)	0.06
Total cholesterol	194.9 (40.6)	208.3 (45.1)	201.3 (4.7)	**0.01**
Triglycerides	141.52 (72.55)	159.08 (95.38)	155.41 (91.27)	0.12
Parity	4.36 (3.02)	3.34 (2.15)	3.19 (2.19)	**<0.001**

Notes: a: Student’s t-test p-value

*n valid; BMI: Body Mass Index; HDL: High Density Lipoproteins; LDL: Low Density Lipoproteins.

Table 3 shows the final model of the logistic regression analysis regarding the outcomes of body (general), thinness and obesity dissatisfaction. Regarding general body dissatisfaction, the variables of education, BMI and physical activity remained related to poor perception. The results showed that with each increase in BMI, women were 1.28 times more likely to be dissatisfied with their bodies (p<0.001). Those with lower education (OR = 3.29) and those who did not practice physical activity (OR = 2.43) were more likely to rate their body perception as poor.

**Table 3:** Final model of logistic regression analysis for the outcomes of body dissatisfaction (general), dissatisfied due to thinness and obesity, Parnamirim, Santa Cruz, RN, Brazil, 2017.

Body dissatisfaction (general)				Thinness dissatisfaction		Obesity dissatisfaction	
Variables		OR	CI 95%	OR	CI 95%	OR	CI 95%
Age	< 60 years	1				1	
	≥ 60 years	1,65	0.93:2.90			1.72	0.93:3.18
p-value		0.08				0.07	
Education	0–3 years	3.29	1.75:6.29			3.91	1.96:7.82
	4 years or more	1				1	
p-value		**<0.001**				**<0.001**	
BMI		1.28	1.19:1.38	0.76	0.64:0.90	1.40	1.28:1.53
p-value		**<0.001**		**0.002**		**<0.001**	
Physical activity	Yes	1				1	
	No	2.43	1.41:4.17			2.53	1.42:4.51
p-value		**<0.001**				**0.002**	
Parity	0–2 children					1	
	3 children or more					1.72	0.93:3.28
p-value						0.07	

Notes: OR: Odds Ratio; CI95%: Confidence interval; BMI: Body Index Mass.

As for dissatisfaction with thinness, only BMI remained in the model, and showed a significant association (OR = 0.76 and p-value: 0.02) with the variable, therefore, at each point of decrease in BMI, the probability of women reporting dissatisfaction with thinness increases by about 24%. Regarding dissatisfaction due to obesity, the results show that those who had a low level of education are 3.9 times more likely to be dissatisfied with their image, similarly, with each increase in BMI, there is a 1.4 times greater chance of being dissatisfied with their image body. Similar to the general dissatisfaction result, not practicing physical activity was shown to be a risk factor for obesity dissatisfaction (OR = 2.53; p-value: 0.02) (Table 3).

## Discussion

This article mainly analyzed the relationship between weight gain and the perception of body image in women in the menopausal transition. It was possible to observe that there was an association between indicator measures and poor perception of body image. As observed in our findings, body image dissatisfaction is a public health concern due to its high prevalence, and because it is often related to worse behavioral habits [7,33].

Comparisons between ideal and perceived figures in women showed that thinness was the most desirable body image among the participants. This was evident as about 80% classified themselves as dissatisfied with being overweight, while, in a similar percentage, overweight and obese also represented more than 80% of the sample [34,35]. This reinforces the internalization of the ideal thinness pattern, which can lead to increased body dissatisfaction in middle-aged women [34–36].

Such results were clear in this study. It was observed that with each increase in BMI, there is a greater chance of women reporting poor body satisfaction, both in general and related to obesity, similar to what was seen by Becker et al., [37]. Previous studies indicate that BMI is strongly associated with high body dissatisfaction in middle-aged women [38,39], which may be due to the lack of acceptance of changes arising from the aging process, in addition to changes caused by menopause [40,41].

Furthermore, middle-aged women have had the tendency of overestimating their body perception [42], as analyzed by Deeks and McCabe [43], who showed that women, when asked to choose a silhouette that best matched their size, marked a larger number than the true one. Therefore, obesity is considered a problem, a characteristic that does not hide the beauty standards imposed by society [44].

Self-assessment of body image is a multidimensional construction that can be influenced by several factors, such as education. It was observed that those who had up to three years of study have a greater chance of being dissatisfied with their body image, especially regarding obesity. Strengthening the hypothesis that the level of education directly influences the body satisfaction of women, the study by Gavin et al., [45] identified that women who had more years of study were more satisfied with their body image. This means that if women were more educated, they could think more positively about their bodies [46]. Therefore, our women, predominantly with low education, may have considered other factors, including cultural aspects at the time of body assessment, thus causing a high prevalence of dissatisfaction.

In relation to age, despite controversial data in the literature, it is also cited as an important factor in the variation of body composition in middle-aged women [8]. However, similar to data reported by Fin et al., [44] and Cameron et al., [8], the findings of this study suggest the non-interference of this variable, since age did not remain in the final logistic regression model.

Middle-aged women are constantly striving to manage their appearance and regain their physical attractiveness to eliminate body-related stress and have self-contentment and psychological stability [47].

According to McLaren and Kuh [48], middle-aged women feel more dissatisfied today than they did at younger ages, including those in their forties. This “turning 50” effect suggests that there is something about being in their fifties that makes most women unhappy with their bodies.

Consequently, this body dissatisfaction has been linked to adverse outcomes such as anxiety and depression among middle-aged women, which can result in an ongoing and potentially dangerous cycle of weight loss and gain associated with numerous negative health effects such as increased blood cholesterol, reduced lean body mass, increased waist-to-hip ratio, high blood pressure, and increased risk of mortality [49–51].In addition, dissatisfaction with body image affects the quality of life and self-esteem of women, promoting body shame and anxiety related to appearance, causing events such as the practice of physical activities to be avoided [50].

In this sense, identifying the factors that, in addition to age and menopausal status (which are not modifiable), contribute to body image distortion is of great relevance. One of the most promising factors in this regard is the practice of physical activity, which is considered a beneficial behavior for maintaining body weight [51].

In this study, the fact of not performing physical activity increased the chance of being dissatisfied with their body image by about two times, both in general and related to obesity. In postmenopausal women, physical activity delays the occurrence of symptoms. However, the frequency of physical activity tends to decrease with increasing age and weight, as obese and older people become less active, either by compromised mobility or by inhibition resulting from dissatisfaction with body image. This causes adverse and lasting impacts on their quality of life, physical health, and cognitive function [52]. Therefore, the practice of physical activity should be stimulated due to its improvement in physical aspects, psychological and social reasons, and thus, improving the quality of life of these women [53].

Another important result is the relationship between parity and the perception of body image. A study carried out in Ceará found that having more than two children increased the chance of being obese by 1.65 times among the women studied [41]. However, our results contradict what is pointed out in the literature, with no significant association between the number of childbirths and body self-image.

The most plausible explanation found is the one that considers the social, educational, and cultural aspects in which women are inserted [10,14]. Since they are a population with low education, there may be less concern with the aesthetic and physical aspects of their body, as a result, the number of children does not influence poor body perception [45], in addition, part of these women still maintains the understanding that parity can be synonymous with health [45].

There are conflicting results regarding the importance that middle-aged women attribute to their body appearance [42]. For some studies [16,36] there is a lower dedication to appearance and a greater appreciation of the functionality of the body, for others [42], older women experience negative feelings and attitudes, such as body dissatisfaction.

### Strengths and limitations

Some limitations of the study should be addressed and, at least, discussed. First, the cross-sectional study design limits the ability to maintain cause-and-effect relationships between factors and outcomes. Second, the convenience sample may limit the ability to extrapolate the data to other populations with similar social profiles. Third, this study did not provide a detailed analysis of menopausal symptoms, which are strongly related to body image dissatisfaction. Finally, the variables that were evaluated by self-report may have been collected with bias, reducing the quality of the data produced. Based on the methodological and statistical rigor adopted, it is expected that these possible limitations have been properly controlled and that the results found, in fact, reflect the real health situation of that population.

On the other hand, the positive points also need to be explored. First, it should be noted that this is one of the first articles to address this issue, simultaneously, in populations in less favored areas and in this age group. Second, this study found a result that can point in a new direction, strengthening the theory that cultural and social values differ from those present in more economically developed societies, impacting women’s health.

## Conclusion

It is concluded that the prevalence of body image dissatisfaction is high in middle-aged and older women, in a sample from the Brazilian Northeast, demonstrating the importance of this theme within the study of the female aging process.

Our data provide scientific support to encourage the use of instruments that assess this public’s body perception, in addition to being able to identify which intrinsic and extrinsic factors are associated with this outcome. In addition, the results found are fundamental so that health professionals involved in body care, especially the female body, can plan health promotion actions aimed at improving the quality of life and, consequently, the body image of these women in this transition stage.

## Supporting information

S1 File (SAV)
